# Sex and age-level differences of walking time in preschool children on an obstacle frame

**DOI:** 10.1186/1880-6805-31-8

**Published:** 2012-04-17

**Authors:** Kosho Kasuga, Shin-ichi Demura, Hiroki Aoki, Sohee Shin, Hiroki Sugiura, Yu Uchida

**Affiliations:** 1 Gifu University, Yanagito, Gifu, Gifu, 501-1193, Japan; 2 Graduate School of Natural Science & Technology, Kanazawa University, Kakuma, Kanazawa, Ishikawa, 920-1192, Japan; 3 Kanazawa College of Art, Kodatuno, Kanazawa, Ishikawa 920-8656, Japan

**Keywords:** Boys, girls, dynamic balance

## Abstract

**Background:**

Stepping over an obstacle is a kind of compound movement that makes walking more difficult, especially for preschool children. This study examines sex and age-level differences in walking time in preschool children on an obstacle frame.

**Methods:**

The participants included 324 healthy preschool children: four-year-old boys (51) and girls (51), five-year-old boys (50) and girls (60), and six-year-old boys (62) and girls (50). A 5 cm- or 10 cm-high obstacle (depth 11.5 cm, width 23.5 cm) was set at the halfway point of a 200 cm × 10 cm walking course.

**Results:**

The participants walked to the end of the course and back as fast as possible under three conditions: no obstacle, low obstacle and high obstacle. Walking time showed age-level differences in all conditions, but there were no differences in sex. Age levels were divided into two groups, with one group within the first six months of their birthday, and the second group within the last six months of that year. Walking time for children in the first half of their fourth year was longer than that of the five- and six-year-old children. In addition, for children in the last half of their fourth year, walking time was longer than both sexes in the last half of their fifth and sixth years. The children in the latter half of their fifth year had a longer walking time in the high obstacle condition than those in the last half of their sixth year. In the four-year-old participants, walking time was shorter with no obstacles than with a high obstacle frame.

**Conclusions:**

In the above data, obstacle course walking time does not show a gender difference, except that the four-year-old participants needed longer than the five- and six-year-old children. Setting the obstacle 10 cm high also produced a different walking time in the five- and six-year-old participants. The high obstacle step test (10 cm) best evaluated the dynamic balance of preschool children.

## Background

Coordination-related nerve function develops remarkably in infancy. Coordination consists of the skillful use of the hands and fingers and the agile movement of the body, while maintaining a balanced posture [1]. Balance is largely divided into static balance and dynamic balance [1]. It is important to evaluate dynamic balance adequately, as it contributes largely to the performance of basic movements. Existing tests evaluating the dynamic balance of preschool children are limited to balance beam walking and line walking tests [2-4]. The former has the risk of an injury, and thus children may be too afraid to perform naturally. The latter is too easy for older children. Hence, it is necessary to develop a test that evaluates adequately and simply the dynamic balance of all preschool children.

Many studies examine gender differences in the dynamic balance of preschool children [3,5,6]. Demura *et al*. [5] reported that no gender differences were found in the line walking test in children aged three to six, and Aoki *et al*. [6] observed similar results in five- and six-year-old children. However, when Demura [3] examined walking times on both the balance beam and the line and a series of hopping time tests on the line and on the stones, only the hopping test showed significant gender differences. Harcherik *et al*. [7] examined gender differences in forward, backward, toe and heel walking on a balance beam in four- to five-year-old, six- to eight-year-old, nine- to eleven-year-old and twelve- to fourteen-year-old children. They reported a difference only in backward walking. The results of the simple line or balance beam walking test, therefore, may not reveal gender differences. Aoki *et al*. [6] showed that stepping over an obstacle is a kind of compound movement that makes walking more difficult, especially for preschool children. Hence, in adjusting to the obstacle, a gender difference may occur in walking time due to greater difficulty.

At the same time, Aoki *et al*. [6] reported that balance beam walking time was significantly longer in five-year-old children than in six-year-old children. Clifton [8] examined age differences in the beam walking test score in two- to five-year-old children, and reported that walking test scores rise with age. According to a study by Demura *et al*. [5], there were no differences in walking time for children three to five years old, but the walking time of the six-year-old children was shorter than that of the five-year-old children. It is thought that the development of dynamic balance affects time walking in a straight line. Obstacles may produce significant age difference in walking time, due to increasing difficulty.

This study examines sex and age-level differences of walking time in preschool children.

## Methods

### Participants

The participants were 324 healthy preschool children, aged four to six years old. Participants' details are shown in Table 1. The experimental purpose and methods were explained to each child and their parents, and their consent was obtained. This study protocol was approved by the Kanazawa University Department of Education.

**Table 1:** Basic statistics of age, height and weight

			Age (years)		Height (cm)		Weight (kg)	
		Number	Mean	SD	Mean	SD	Mean	SD
	4 years (First)	23	4.2	0.2	100.9	3.4	16.5	1.2
	4 years (Last)	28	4.7	0.1	102.4	4.4	16.7	1.6
Boys	5 years (First)	23	5.2	0.1	107.2	4.0	18.0	1.5
	5 years (Last)	27	5.7	0.1	107.4	4.4	17.7	1.8
	6 years (First)	35	6.2	0.1	112.8	5.0	20.1	2.3
	6 years (Last)	27	6.7	0.1	115.9	3.7	20.8	1.5
	4 years (First)	24	4.2	0.2	99.3	4.6	16.0	2.0
	4 years (Last)	27	4.8	0.2	102.6	3.8	16.6	1.9
Girls	5 years (First)	32	5.2	0.1	105.4	3.6	17.7	1.7
	5 years (Last)	28	5.7	0.2	108.2	5.6	18.4	2.9
	6 years (First)	26	6.2	0.2	112.3	3.2	19.7	2.0
	6 years (Last)	24	6.7	0.1	116.2	3.5	21.0	1.7

SD: standard deviation

Results of two-way ANOVA, *P < 0.05

[Height] Age F_1 _= 105.53* (5, 312), Gender F_2 _= 0.96 (1, 312), Interaction F_1 × 2 _= 0.84 (5, 312). Post hoc. Boy, Girl: 4 years < 5 years < 6 years

[Weight] Age F_1 _= 46.74* (5, 312), Gender F_2 _= 0.05 (1, 312), Interaction F_1 × 2 _= 0.76 (5, 312). Post hoc. Boy: 4 years, 5 years < 6 years; Girl: 4 years (First) < 5 years, 6 years; 4 years (Last) < 5 years (Last), 6 years, 5 years (First) < 6 years; 5 years (Last) < 6 years (Last)

### Procedures

**Figure 1:**
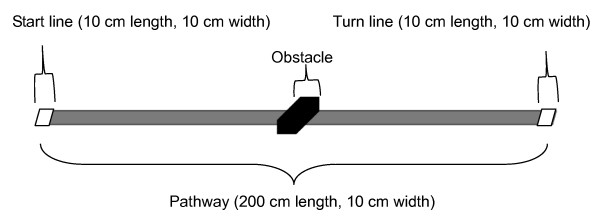
**Course illustration**.

An obstacle (11.5 cm length, 23.5 cm width) was set at the halfway point of a pathway (200 cm length, 10 cm width) (Figure 1). The participants walked along the pathway under three conditions: no obstacle; low (5 cm high); and high obstacle (10 cm high). The participants began walking at a start line (10 cm × 10 cm tape line), reached the turn line (10 cm × 10 cm tape line), and returned to the start line. The participant's time from the start to the finish was measured.

The children were instructed to walk as fast as possible, to change direction quickly after reaching the turn line, and to return to the original position. We started the measurement again if one child stepped out of the line or touched an obstacle. If this occurred three times in succession, measurement was judged to be impossible.

All of the children could complete the no obstacle test, but four children could not finish the course under the low and high obstacle conditions. The walking time was measured with a stopwatch (Seiko, SO31-4000).

### Data analysis

The intraclass correlation coefficient for each test was calculated to examine trial-to-trial reliability. Pearson's correlation coefficient was calculated to examine the relationship between physique (height and weight) and walking time. A two-way analysis of variance (ANOVA) was used to test mean differences among the gender and age-level for height and weight. A three-way ANOVA was used to test mean differences between age, gender and obstacle conditions for each walking time. Age levels were further divided into two groups, with the one group within the first six months of their birthday, and the second group within the last six months of that year. When showing a significant interaction or a significant main effect, Tukey's Honestly Significant Difference test was used for multiple comparisons. The level of significance was set at *P <*0.05.

## Results

The intraclass correlation coefficient of the walking time for each condition was 0.62 to 0.76 in four-year-old boys, 0.71 to 0.81 in four-year-old girls, 0.64 to 0.83 in five-year-old boys, 0.69 to 0.82 in five-year-old girls, 0.66 to 0.78 in six-year-old boys and 0.60 to 0.72 in six-year-old girls.

Table 1 shows the basic statistics for age, height and weight, and the results of the two-way ANOVA (age × gender). An insignificant interaction was found between height and weight. Height and weight generally tended to increase with age.

The results of correlations and partial correlations between physique and each walking time, according to age and sex, are shown in Table 2. Correlations below 0.43 between walking time and physique were found to be significant, except for girls in the no obstacle condition. Partial correlations were below than 0.35 and showed no significance with respect to the high obstacle course.

**Table 2:** Correlations and partial correlations considering age-effect between physique and each walking time, according to gender

		Boys		Girls	
		Height	Weight	Height	Weight
**Correlations**	No obstacle condition	-0.38*	-0.29*	-0.25*	-0.14
	Low obstacle condition	-0.38*	-0.34*	-0.29*	-0.19*
	High obstacle condition	-0.43*	-0.39*	-0.33*	-0.23*
**Partial correlations**	No obstacle condition	-0.35*	0.30*	0.16*	0.17*
	Low obstacle condition	0.14	-0.28*	0.18*	0.16*
	High obstacle condition	-0.08	-0.09	0.12	0.06

*P < 0.05

The basic statistics regarding walking time according to age, gender and obstacle, and the results of the three-way ANOVA (age × gender × obstacle) are shown in Table 3. An insignificant interaction was found between all conditions, and a significant main effect was found in age and obstacle type. Multiple comparisons showed that in the no obstacle condition children performed slower during the first half of their fourth year than all of the five- and six-year-old participants. Walking time was also slower in the older half of the four-year-old group than in the older half of the five- and the six-year-old boys and girls. In the low obstacle course, the children in the first half of their fifth year were slower than boys in the older half of their sixth year. The no obstacle course obtained the same results, for both sexes. In the high obstacle course, in addition to yielding the same results as the no obstacle course for both sexes, it was shown that the younger half of the five-year-old group was slower than the older half of the six-year-old group. Moreover, a significant difference among conditions was found only between the two four-year-old groups, and the no obstacle course proved to be slower than the high obstacle course.

**Table 3:** Walking time in seconds by age, gender and obstacle

		No obstacle		Low obstacle		High obstacle					
						
		Mean	SD	Mean	SD	Mean	SD	F-value			Post hoc
	4 years (First)	6.0	2.0	6.5	2.4	7.5	4.1	F1 (1,307) = 0.65	No obstacle condition	Boy•Girl	4 years (First) > 5 years (First), 5 years (Last), 6 years (First), 6 years (Last)
	4 years (Last)	5.5	1.3	5.9	1.3	6.7	1.9	F2 (5,307) = 27.93*		Boy•Girl	4 years (Last) > 5 years (Last), 6 years (First), 6 years (Last)
					
Boys	5 years (First)	4.7	1.5	5.3	2.0	5.3	0.9	F3 (2,614) = 59.58*	Low obstacle condition	Boy•Girl	4 years (First) > 5 years (First), 5 years (Last), 6 years (First), 6 years (Last)
	5 years (Last)	4.2	0.9	4.6	1.3	4.9	1.1	F4 (5,307) = 0.17		Boy•Girl	4 years (Last) > 5 years (Last), 6 years (First), 6 years (Last)
					
	6 years (First)	4.3	0.8	4.5	1.0	4.8	1.1	F5 (2,614) = 0.26		Boy	5 years (First) > 6 years (Last)
	6 years (Last)	3.7	0.6	3.9	0.9	4.2	1.1	F6 (10,614) = 2.72*	High obstacle condition	Boy•Girl	4 years (First) > 5 years (First), 5 years (Last), 6 years (First), 6 years (Last)
				
	4 years (First)	5.9	2.1	6.8	2.7	7.2	2.4	F7 (10,614) = 0.44		Boy•Girl	4 years (Last) > 5 years (Last), 6 years (First), 6 years (Last)
	4 years (Last)	5.8	1.5	6.1	1.7	6.9	2.5			Boy•Girl	5 years (First) > 6 years (Last)
			
Girls	5 years (First)	4.9	1.4	5.3	1.4	5.5	1.3		4 years	Boy•Girl	High obstacle condition > No obstacle condition
	5 years (Last)	4.3	0.8	4.8	1.0	4.9	1.0				
					
	6 years (First)	4.3	1.1	4.4	0.8	4.8	0.9				
	6 years (Last)	4.2	1.2	4.3	1.0	4.4	1.0				

F1: gender; F2: age; F3: condition; F4: interaction (gender × age); F5: interaction (gender × condition); F6: interaction (age × condition); F7: interaction (gender × age × condition); SD: standard deviation. *P < 0.05.

**Figure 2:**
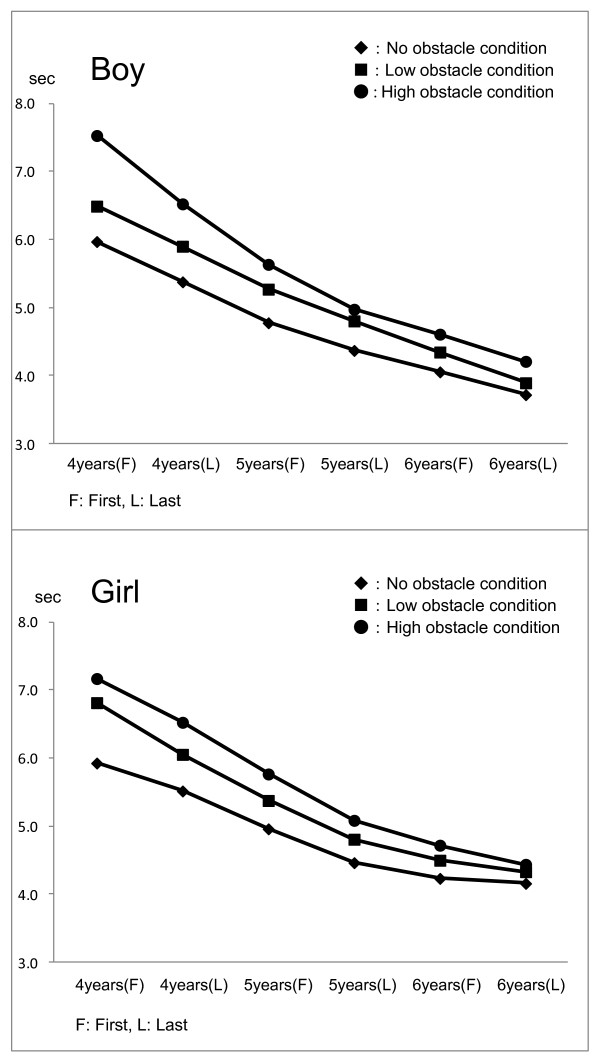
**Moving means of walking times according to each condition in boys and girls**.

Figure 2 shows moving means of walking times according to each condition in boys and girls. Each walking time shortens with age and its degree showed a small tendency after the last half of 5 years old in boys and girls.

## Discussion

If a person is taller, they will also generally have long legs and long steps. Hence, with taller children, walking time should be shorter across a constant distance. The results show that the relationship between height and walking time was significant in the no obstacle condition for boys and in the no obstacle and low obstacle conditions for girls, but both had values lower than 0.35. Adding an obstacle to the course [6] requires a compound stepping movement. It is agreed that stepping affects walking time significantly. In the case of preschool children, even with a 10 cm tall obstacle, it is necessary to elevate the knee to step over it. Hence, it is thought that this unstable motion results in a longer walking time. However, the present results show that the obstacle height had very little effect on the walking time.

Walking time showed an insignificant gender difference in all conditions, for all ages (F-value = 0.65). Demura *et al*. [5] reported that gender differences were not found in the walking time of three- to six-year-old children. When setting an obstacle, difficulty during walking increases even on a level path. Hence, it was thought that a gender difference would occur in walking time; however, this hypothesis was rejected. Walking is one of the most basic human movements. Therefore, it is inferred that girls could perform similarly to boys, even if a task like stepping over an obstacle was added.

Preschool children, particularly children under three years old, are unstable when walking on a thin line. Their postural stability requires greater balance ability. Hence, the line walking test, which is more difficult than normal walking, has been used in many studies [3,5] to evaluate dynamic balance. In addition, when stepping over an obstacle, balance ability demands a momentary one-legged standing position. It is assumed, therefore, that preschool children with superior dynamic balance can step smoothly over an obstacle and achieve other ambulatory tasks in a shorter time.

Significant age differences exist in walking time, regardless of the obstacle (F-value = 27.93). Figure 2 shows the average walking time, which has the tendency to shorten generally and decrease with age, especially after the last half of the fifth year. According to Niederer *et al*. [9], step number on a balance beam increases over the nine months in five-year-old children. In this study, due to space limitations, a frame (10 cm) in place of a line (2 cm) [5] was used, and an obstacle was added. The present results reveal that the walking time shortens remarkably across age levels up to the second half of the fifth year, whether or not the obstacle is present. In older children, walking time does not change significantly. Although walking on a line has been used to evaluate the dynamic balance of preschool children [5], the present results suggest that it may not be adequate for use in children aged five years and older.

The walking time differed between the no-obstacle and high obstacle conditions only in four-year-old boys and girls (F-value = 59.58). When stepping over an obstacle, children must lift one foot higher than the obstacle [6]. It is necessary to maintain a stable posture, or dynamic balance, because walking necessarily involves standing on only one leg. It may be difficult for four-year-old children to keep a stable posture on one leg due to their short height, even with obstacles at a height of 10 cm. Therefore, it is thought that they need a longer time to step over an obstacle. In addition, no significant difference was found in the walking time between the no-obstacle and the high obstacle conditions in participants five years old and older. Hence, in ages five and over, because of their height (over 105 cm) and the development of their dynamic balance, it is inferred that an obstacle of 10 cm height was not a limitation to walking. Takagi [10] reported that achievement of compound movement enables until 6 years old. In this study, walking along the obstacle frame requires a compound movement of walking and stepping over. Because they are basic movements, it is inferred that five- to six-year-old children can easily pass the obstacle course.

Because even four-year-old children can easily achieve simple walking tasks, including a high obstacle (10 cm) on a path may be useful to evaluate dynamic balance when considering all preschool children, which includes both five- and six-year-olds.

## Conclusion

There is no gender difference in walking time after age four, regardless of the obstacle. Walking time shortens with age, but differs little after the last half of the fifth year, except with the high obstacle. A high obstacle affects walking time in four-year-old children. A high obstacle equips the frame to better evaluate dynamic balance in walking.

## Competing interests

The authors declare that they have no competing interests.

## References

[B1] Demura S Text book of Health and Sports Science 2005 Tokyo: Kyorin-Shoin 38 60 In Japanese

[B2] Demura S Contribution of physical fitness and throw form to ball-throw distance and the sex difference in preschool children Japan J Phys Educ Hlth Sport Sci 1993 37 339 350 In Japanese with English abstract

[B3] Demura S Development and sexual difference of static and dynamic balance in preschool children Japan J Phys Educ Hlth Sport Sci 1995 40 67 79

[B4] Bürgi F Meyer U Granacher U Schindler C Marques-Vidal P Kriemler S Puder JJ Relationship of physical activity with motor skills, aerobic fitness and body fat in preschool children: a cross-sectional and longitudinal study (Ballabeina) Int J Obes (Lond) 2011 35 937 944 10.1038/ijo.2011.54 21448128

[B5] Demura S Nagasawa Y Kasuga K The development of dynamic balance and its sex difference in preschool children 1994 39 Journal of Medical Education 368 376

[B6] Aoki H Demura S Kasuga K Shin S Kawabata H Examining difference in walking time on a balance beam with an obstacle based on gender and age in preschool children Japanese Society of Education and Health Science 2011 56 352 355 In Japanese with English abstract

[B7] Harcherik DF Carbonari CM Cohen DJ Attentional and perceptual measures: developmental changes Schizophr Bull 1982 8 349 355 7112045 10.1093/schbul/8.2.349

[B8] Clifton MA Effects of special instruction and practice by preschool age children on performance of object projection and stability tests Percept Mot Skills 1978 47 1135 1140 10.2466/pms.1978.47.3f.1135 745887

[B9] Niederer I Kriemler S Gut J Hartmann T Schindler C Barral J Puder JJ Relationship of aerobic fitness and motor skills with memory and attention in preschoolers (Ballabeina): a cross-sectional and longitudinal study BMC Pediatr 2011 11 11 34 10.1186/1471-2431-11-11 21569343 PMC3107157

[B10] Takagi N Exercise play by preschool children/latest edition 2009 Tokyo: Fumaidou-Syuppan 50 64 In Japanese

